# Identification of a Novel, Small Molecule Partial Agonist for the Cyclic AMP Sensor, EPAC1

**DOI:** 10.1038/s41598-017-00455-7

**Published:** 2017-03-22

**Authors:** Euan Parnell, Stuart P. McElroy, Jolanta Wiejak, Gemma L. Baillie, Alison Porter, David R. Adams, Holger Rehmann, Brian O. Smith, Stephen J. Yarwood

**Affiliations:** 1 Institute of Molecular, Cellular and Systems Biology, College of Medical Veterinary and Life Sciences, University of Glasgow, Glasgow, G12 8QQ UK; 2 European Screening Centre, University of Dundee, Biocity Scotland, Newhouse, ML1 5UH UK; 3 Institute of Biological Chemistry, Biophysics and Bioengineering, Heriot-Watt University, Edinburgh Campus, Edinburgh, EH14 4AS UK; 4 Institute of Chemical Sciences, Heriot-Watt University, Edinburgh Campus, Edinburgh, EH14 4AS UK; 5 Department of Molecular Cancer Research, Centre of Biomedical Genetics and Cancer Genomics Centre, University Medical Centre Utrecht, Utrecht, The Netherlands

## Abstract

Screening of a carefully selected library of 5,195 small molecules identified 34 hit compounds that interact with the regulatory cyclic nucleotide-binding domain (CNB) of the cAMP sensor, EPAC1. Two of these hits (I942 and I178) were selected for their robust and reproducible inhibitory effects within the primary screening assay. Follow-up characterisation by ligand observed nuclear magnetic resonance (NMR) revealed direct interaction of I942 and I178 with EPAC1 and EPAC2-CNBs *in vitro*. Moreover, *in vitro* guanine nucleotide exchange factor (GEF) assays revealed that I942 and, to a lesser extent, I178 had partial agonist properties towards EPAC1, leading to activation of EPAC1, in the absence of cAMP, and inhibition of GEF activity in the presence of cAMP. In contrast, there was very little agonist action of I942 towards EPAC2 or protein kinase A (PKA). To our knowledge, this is the first observation of non-cyclic-nucleotide small molecules with agonist properties towards EPAC1. Furthermore, the isoform selective agonist nature of these compounds highlights the potential for the development of small molecule tools that selectively up-regulate EPAC1 activity.

## Introduction

The exchange protein activated by cAMP (EPAC) isoforms, EPAC1 and EPAC2, are guanine nucleotide exchange factors (GEFs) for the Ras GTPase homologues, Rap1 and Rap2, which they activate independently of the classical cAMP-sensing enzyme, protein kinase A (PKA)^1^. As such, EPACs represent a novel means through which the ubiquitous second messenger, cAMP, can exert control over cell activity.

We have previously defined a role for EPAC1 in cAMP mediated, PKA-independent transcriptional induction of the suppressor of cytokine signalling 3 (SOCS3) gene, which occurs through the activation of C/EBP transcription factors in vascular endothelial cells (VECs)^2, 3^. Classically, SOCS3 induction occurs in response to inflammatory cues, such as interleukin 6 (IL6) stimulation, with subsequent activation of the JAK-STAT signalling pathway^4^. SOCS3 is then able to bind to JAK-phosphorylated receptors *via* the SOCS3 SH2 domain, inhibiting the activation of STATs 1 and 3 by JAK^5^. Furthermore, SOCS3 is able to direct the proteasomal degradation^5^ of various proteolytic targets, including JAK2^6^, resulting in a negative feedback loop that attenuates inflammatory signalling from the IL-6 receptor^2, 7, 8^.

Recent work has suggested that EPAC1 and SOCS3 may also play a key role in the central control of energy balance. For example, leptin has also been shown to induce SOCS3 expression in INS-1 β-cells and human pancreatic islets *in vitro* and in the pancreatic islets of obsese, ob/ob, mice *in vivo* ^9^, where it inhibits STAT3-dependent rat preproinsulin 1 gene promoter activity^9^. Taken together, these observations suggest that there may be a link between SOCS3 induction and the development of type 1 and type 2 diabetes (T2D). Indeed, it has been shown that SOCS3 may be involved in the development of insulin resistance, normally associated with T2D. This it thought to occur through the disruption of insulin receptor (IR) signalling to phosphoinositide 3-kinase (PI3K) and the mitogen activated protein kinase, ERK, through its key intracellular substrate, insulin receptor substrate 1 (IRS-1)^10^. In this case, increased SOCS3 levels inhibit IR tyrosine phosphorylation on Tyr 960, thereby preventing coupling to IRS-1^11^. IRS-1 is then targeted for proteosomal degradation thereby preventing down-stream signalling to PI3K and ERK and preventing the metabolic actions of insulin^12, 13^.

Clearly, the potential for EPAC1/SOCS3 signalling to regulate inflammation^7, 8^ and leptin^14, 15^ and insulin signalling indicates that EPAC1 may be a valid drug target for a range of disease states^16^. Indeed, this has prompted a number of laboratories to use high throughput screening (HTS) of compound libraries to search for small molecule regulators of EPAC activity^17–20^. Arguably, the most successful of these HTS strategies involved the use of the fluorescent cAMP analogue, 8-NBD-cAMP, to screen for competitive inhibitors of full-length EPAC2, leading to the discovery of the EPAC2-selective inhibitor, ESI-05 (4-Methylphenyl-2,4,6-trimethylphenylsulfone)^21^. Similarly, a non-selective EPAC1 and EPAC2 inhibitor, ESI-09 (3-(5-tert-butyl-isoxazol-3-yl)-2-[(3-chloro-phenyl)-hydrazonol]-3-oxo-propionitrile), was identified by similar protocols^22–24^, however, concerns have been raised about the mechanisms of action of this compound, which has been suggested to display non-specific protein denaturing properties^25^. Interestingly, HTS using 8-NBD-cAMP competition assays has been limited to screens involving EPAC2, likely due to the limited fluorescence of 8-NBD-cAMP when bound to the cyclic nucleotides binding domain (CNB) of EPAC1 compared to EPAC2^19^. This difference may be linked to structural differences between EPAC1 and EPAC2 within the CNB that influences the selective docking of ligands to the cAMP-binding pocket^26^. Both uncompetitive (CE3F4) and non-competitive EPAC1 inhibitors (5225554 and 5376753) have also been identified through HTS using an *in vitro* EPAC1 GEF activity assay^18^ and an EPAC-based bioluminescence resonance energy transfer-based assay^17^, respectively. Notably, none of these HTS approaches has isolated small molecule agonists of EPAC activity, the identification of which would provide important tools to probe the mode of action of EPAC in multiple disease states.

In the current study, we used the isolated CNBs of EPAC1 and EPAC2 to develop a robust 8-NBD-cAMP competition assay to identify compounds that interact with EPAC1. This is the first report of the use of EPAC1 in HTS and the success of the approach was confirmed by the identification of novel ligands (I942 and I178) with partial agonist activity towards EPAC1, but not EPAC2. To the best of our knowledge, this compound represents the first non-cyclic nucleotide ligand to display agonist properties towards EPAC proteins. Furthermore, the potential to activate EPAC1 activity, independently of EPAC2, may facilitate the development of effective EPAC1-targetted therapeutic agents. We therefore identified a novel experimental tool to investigate the role of EPAC1 in health and disease.

## Results

### High Throughput Screening (HTS) of Small Molecular Regulators of EPAC1

A fluorescence-based HTS assay based on the displacement of the fluorescent cAMP analogue, 8-NBD-cAMP, from full-length, recombinant EPAC2, has proven to be an effective method for the identification of EPAC-selective small molecule antagonists of EPAC activity^21^. Our objective was to modify this approach to identify new regulators of EPAC1 activity. In order to develop the 8-NBD-cAMP competition assay for HTS of EPAC1, the isolated cyclic nucleotide-binding domain (CNB) of EPAC1 was used, since this fragment contains the key cAMP-regulated, activation domain for EPAC1 and displays greater solubility compared to full-length recombinant EPAC1^27^. We therefore carried out large-scale recombinant protein purification of the CNBs of EPAC1 (amino acids 169–314) and EPAC2 (amino acids 304–453, incorporating the functional second CNB of EPAC2), resulting in the production of soluble, 50 kDa proteins (Supplemental Figure 1) corresponding to either GST-EPAC1-CNB (EPAC1-CNB) or GST-EPAC2-CNB (EPAC2-CNB).

**Figure 1:**
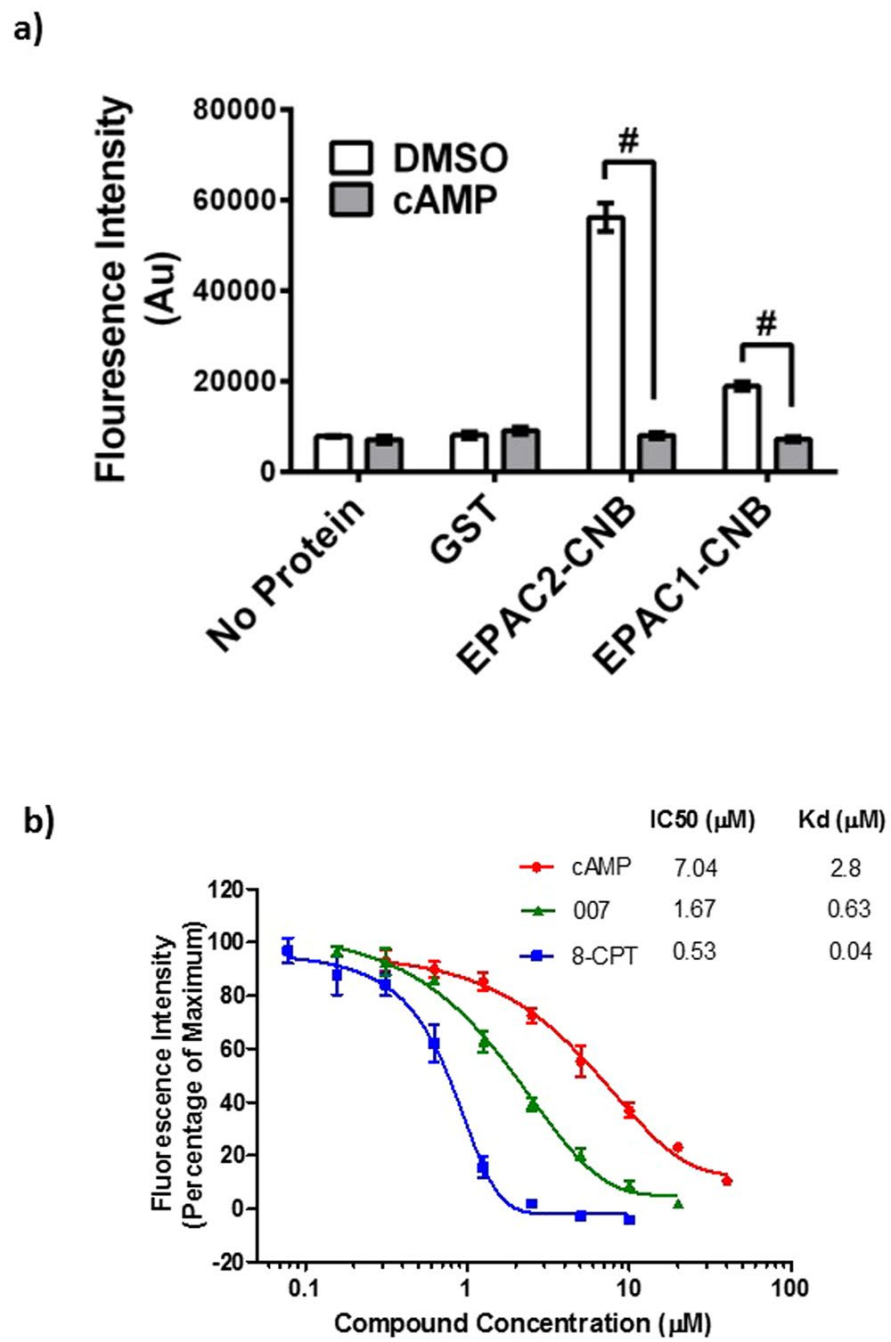
8-NBD-cAMP Competition Assay Using Purified EPAC1-CNB and EPAC2-CNB Proteins. (**a**)The 8-NBD-cAMP competition assay was carried out as previously described^19^. Briefly, purified EPAC1-CNB (0.8 µM), EPAC2-CNB (0.8 µM), GST (0.8 µM) or assay buffer (no protein) was incubated in the presence of cAMP (50 µM) or DMSO. 8-NBD-cAMP (0.1 µM) was then added to every well and the fluorescence intensity was measured after four hours. Significant inhibition of fluorescence in the presence of cAMP is indicated, ^#^P < 0.0001 (n = 3). (**b**) Inhibition of 8-NBD-cAMP (62.5 nM) fluorescence, resulting from interaction with EPAC1-CNB (0.8 µM), is plotted in the presence of varying concentrations of known competitor compounds; cAMP (red squares), 007 (blue circles) or 8-CPT (green triangles). IC50 values were calculated for each competition assay and are shown in comparison with published Kd values for each competitor.

To validate the folding and suitability of EPAC-CNBs for HTS, we incubated either GST, EPAC1-CNB or EPAC2-CNB with 8-NBD-CAMP and measured the resulting fluorescence intensities (485/515 nm, ex/em). In agreement with published data^27^, the fluorescence produced by 8-NBD-cAMP was significantly increased in the presence of EPAC1-CNB and EPAC2-CNB, with no change observed with GST alone or in the absence of protein (Fig. 1a). Therefore, changes in 8-NBD-cAMP occur as a direct result of interaction with either EPAC-CNB and not with the GST tag. However, as described previously for full length, recombinant EPAC2^21^, EPAC2-CNB promoted a greater increase in fluorescence (6.94 fold) than EPAC1-CNB (2.62 fold, Fig. 1a). The change in fluorescence was considerably lower than previously described in the presence of the full length EPAC2 protein, although is consistent with previous reports using the isolated CNB of EPAC2^19^. We suggest that the isolated CNBs display intrinsic disordered characteristics, altering the protein-probe complex stability or the hydrophobic environment favourable to 8-NBD-cAMP fluorescence. To confirm that increases in 8-NBD-cAMP fluorescence were a direct result of a specific interaction, the fluorescence intensity was measured in the presence or absence of saturating concentrations of cAMP. We found that incubation of EPAC-CNBs with cAMP (1 mM) reduced 8-NBD-cAMP fluorescence to basal levels due to the displacement of 8-NBD-cAMP from the hydrophobic cAMP binding pockets of EPAC1 and EPAC2 CNB, indicating a competitive mode of binding (Fig. 1a). To optimise protein-ligand interactions and increase fluorescence intensity for the EPAC1-CNB assay we varied buffer pH and ionic strength of the assay buffer (Supplementary Figure 2a) and determined optimal concentrations of both EPAC1-CNB protein and 8-NBD-cAMP probe to use with these optimised assay buffer conditions (Supplementary Figure 2b). The lowest concentrations of protein (0.8 µM) and 8-NBD-cAMP (62.5 nM to 0.5 µM) which produced a minimum three fold S/B were selected in order to maximise hit detection (Supplementary Figure 2b). We also carried out quality control tests using these optimised conditions and found that the EPAC1-CNB competition assay displayed excellent stability up to 72 hours after initial mixing of protein and probe and a minimum incubation period of four hours was chosen to ensure a stable signal was observed and minimise well variability (Supplementary Figure 3a). The assay was stable in the presence of DMSO concentrations under 1% (v/v), as normally required for compound library screening (Supplementary Figure 3b). In addition, intra- and inter-384-well plate variability was low, indicating high reproducibility within and between plates (Supplementary Figures 4 and 5). Under these conditions, a probe concentration of 62.5 nM resulted in IC_50_ values that most accurately reflected the published affinities of each compound (Fig. 1b)^28^. The inhibitory profiles of cAMP, 007 and 8-CPT (Fig. 1b) reveal that this probe concentration allows the identification of competitor compounds with a relatively broad range of affinities.

### HTS of Small Molecule Competitors of 8-NBD-cAMP-binding to the EPAC1-CNB

**Figure 2:**
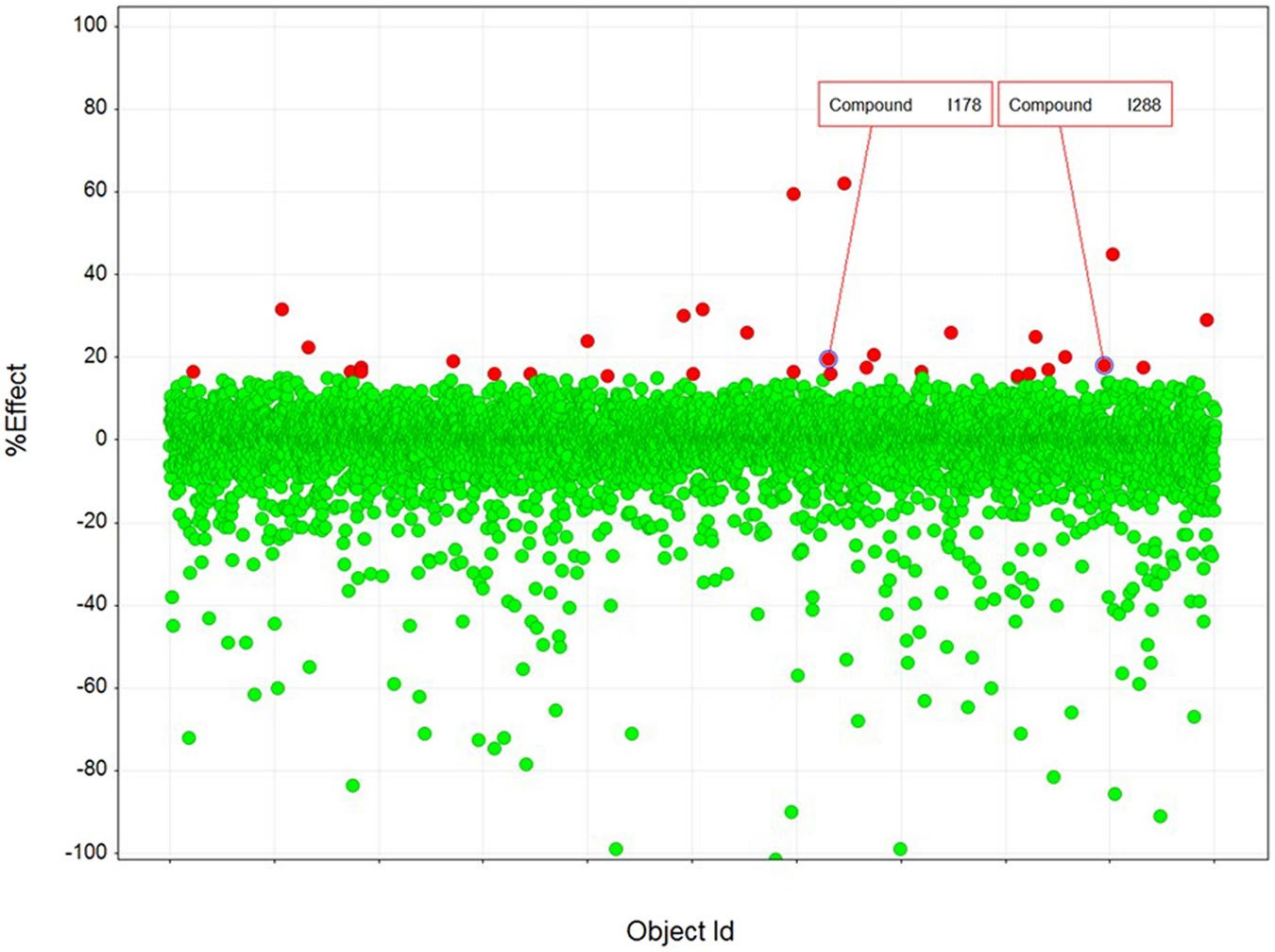
Single Point Testing for Compounds Capable of Disrupting. Interaction between EPAC1-CNB and 8-NBD-cAMP in HTS Compounds from the BioAscent library of diverse lead like structures were assessed in 384 well plates at 10 µM with 0.25% DMSO final, n = 1 over two testing days. Responses (% effect) from each compound tested are shown. For the screen a selection cut-off of 15% was calculated using the median plus three times the robust standard deviation (scaled median absolute deviation) of the percentage inhibition across all compound wells, generating 33 hits (indicated with red dots) and a 0.67% hit rate. The screen performed extremely well with consistent levels of maximum (Max) and minimum (Min) control responses, Z’ values and S/B ratios. %CV values were calculated for matching control wells as described (materials and methods) and were below 10%, indicating excellent inter-plate and inter-day variability.

**Figure 3:**
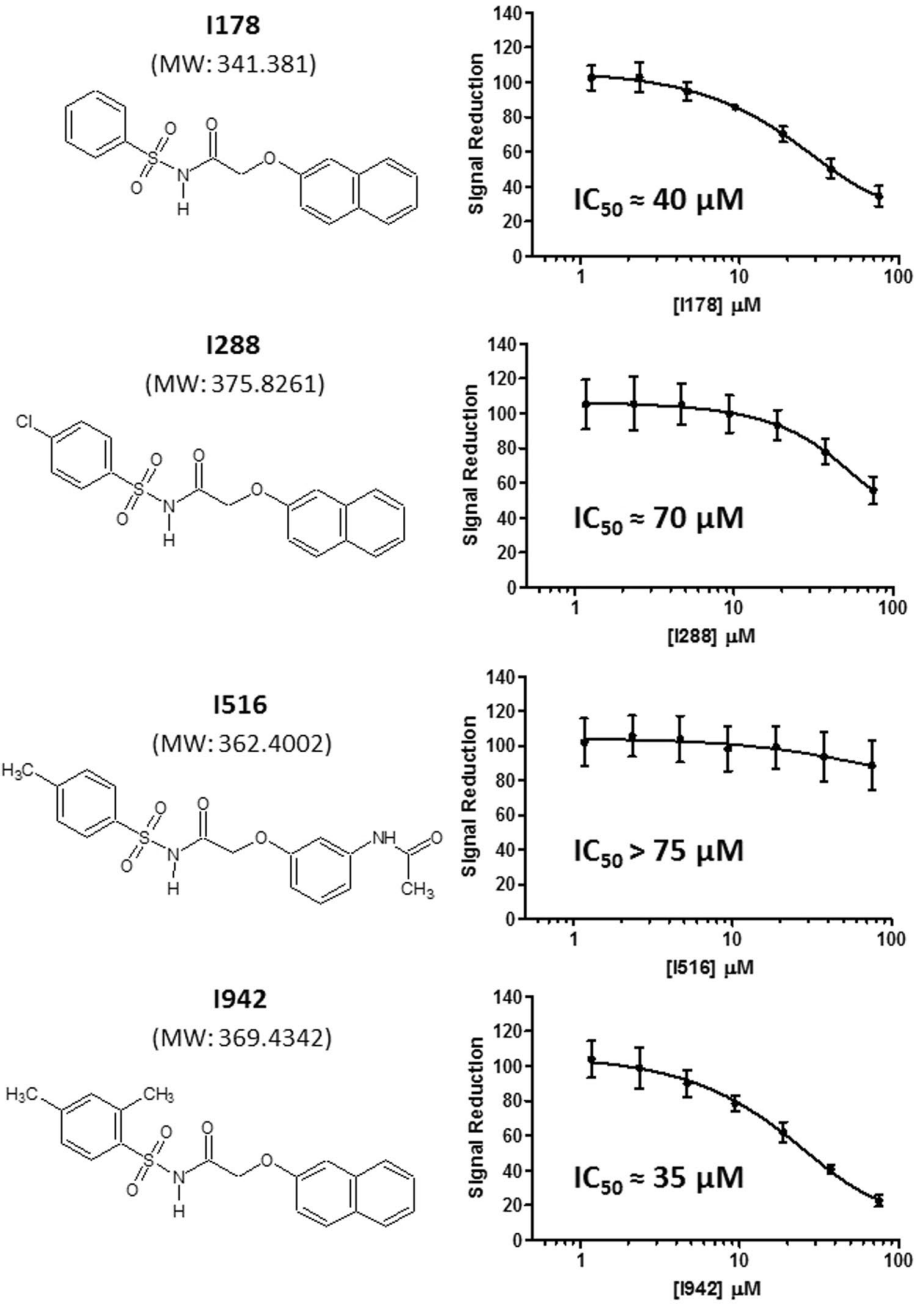
Dose Response Testing of Hits against EPAC1-CNB. The three hit compounds isolated from the Bioascent libraries (structures and molecular weights are shown on the *left*) were tested in a 7-point dose response format (75 µM top concentration, 1 in 2 serial dilution, n = 6 on one plate) alongside a non-binding compound (I516) in the EPAC1-CNB, 8-NBD-cAMP binding assay with final DMSO concentration of 0.75%. IC_50_ values are shown in the individual graphs.

Having optimised assay conditions for EPAC1-CNB HTS, 5195 small molecules from the commercially available BioAscent Compound Cloud of diverse lead like structures were screened at 10 µM, to identify novel EPAC1-interacting compounds. The screen performed well, displaying acceptable signal and variability throughout with consistent maximum and minimum control responses, Z’ values ranging from 0.63 to 0.83 and S/B ratios from 4.5 to 4.9 (Fig. 2). All percentage coefficient of variance (%CV) values were below 7%, indicating satisfactory inter-plate and inter-day variability. Using a 15% inhibition cut-off for 8-NBD-cAMP fluorescence in the assay, the screen produced a 0.64% hit rate and identified 33 compounds as potential inhibitors of 8-NBD-cAMP binding to the EPAC1-CNB (Fig. 2; red dots). Any compounds showing values significantly below the x-axis (>3 SD below) in Fig. 2 are very likely to be fluorescent in the 8-NBD-cAMP competition assay. The 33 potential hits were then tested in the EPAC1-CNB binding assay using a 7-point, 1 in 3 dilution series, with a 30 µM maximum compound concentration, and a final DMSO concentration of 0.75% (v/v). Two compounds were confirmed as demonstrating effective reduction of the fluorescence signal (I288 and I178; Fig. 2) and were used to identify a further 32 structurally related compounds from the larger BioAscent library of 120,000 compounds (results not shown). These compounds were then tested for potency in dose response format (30 µM top concentration and final DMSO concentration of 0.5%). This screen identified a further compound, I942, that, similar to I288 and I178, produced a dose-dependent reduction (75 µM top concentration) of 8-NBD-cAMP fluorescence in the EPAC1-CNB assay (Fig. 3). Moreover, neither I942 nor I178 displayed auto-fluorescence in the range of concentrations used in these assays (Supplementary Figure 6). Thus, the relative IC_50_ values measured were approximately 70 μM, 40 μM and 35 μM, for I178, I288 and I942 respectively, compared to an IC_50_ of around 4 μM, for cAMP under the same conditions (Fig. 1b). In these experiments, a non-active compound, I516, served as a negative control and produced an IC_50_ value of greater than 70 μM. These results suggest that I178, I288 and I942 competitively inhibit 8-NBD-cAMP binding to the EPAC1-CNB, with I942 proving to be the most potent competitor.

### Effects of I942 and I178 on EPAC1 and EPAC2 GEF activity *in vitro*

**Figure 4:**
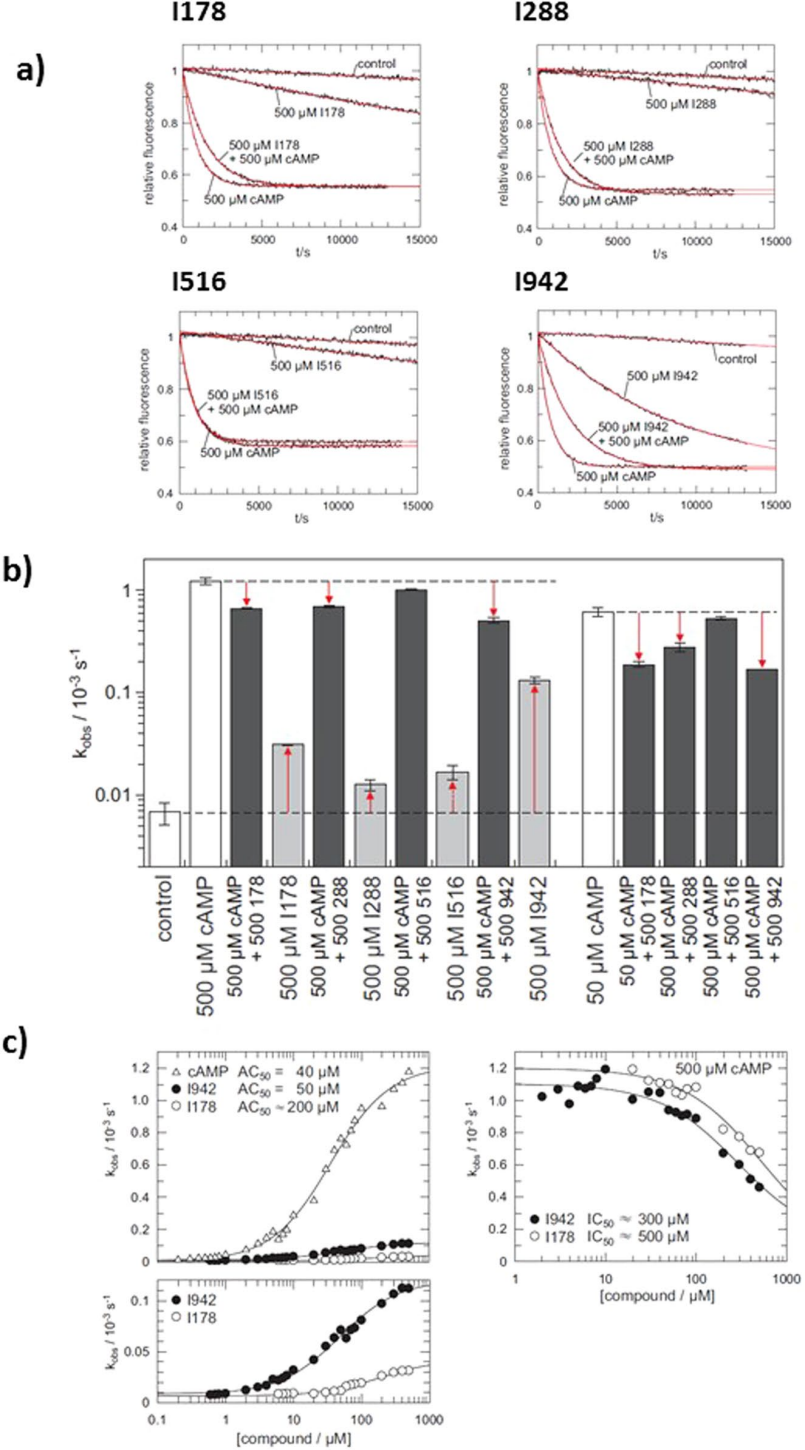
GEF activity of EPAC1. (**a**) Time trace of the exchange reaction with 500 μM cAMP either 500 μM I178, I288, I516 or I942 where indicated. The data are fitted as single exponential decay with off-set (red lines) to obtain the rate constants k_obs_, as shown in (**b**). Rate constants from exchange reactions with either 500 μM or 50 μM cAMP, the presence or absence of 500 μM I178, I288, I516 or I942, as indicated, are presented as a bar graph (n = 4). (**c**) Exchange activity induced by cAMP, I942 or I178. The dependency of k_obs_ on the concentration of cAMP (open triangles), I942 (closed circles) or I178 (open circles) is plotted on the left. The lower panel shows a magnification of the I942 and I178 induced activity. Inhibition of cAMP induced exchange activity by I942 and I178 is shown on the right. k_obs_ were determined in the presence of 500 μM cAMP and various concentrations of I942 or I178.

We next characterised the effects of I178, I288, I516 and I942 on EPAC catalytic activity *in vitro*. For this, we used the well-characterised EPAC GEF activity assay^29^. The assay is based on the EPAC-stimulated dissociation of fluorescent MANT-GDP from recombinant Rap1 in the presence of excess non-fluorescent nucleotide. Addition of 500 μM cAMP promoted a robust increase in EPAC1 activity, demonstrated by an accelerated decay in MANT-GDP signal over time. In agreement with the 8NBD-cAMP competition assays (Fig. 3), cAMP-induced EPAC1 activity was reduced in the presence of I942 and, to a lesser extent, I178 and I288 (Fig. 4a). The inhibitory effect of I288 was weaker than that of I178. I516 has no detectable inhibitory effect. To examine further the weak inhibitory effects of I178 and I288, activity assays were carried out using 500 μM I178 and I288 in the presence of 50 μM cAMP (Fig. 4b). Under these conditions, EPAC1 is not fully saturated with cAMP and thus the cAMP induced activity is weaker as observed with 500 μM, as used in Fig. 4a. With the inhibitors being applied at 10 fold higher concentration than cAMP, inhibition of EPAC1 was also observed for I178 and I288, despite their weaker affinities (Fig. 4b).

Interestingly, in the absence of cAMP, I178, I288 and, in particular, I942 also induced EPAC1 activity (Fig. 4a and b). To explore this further, the dependency of EPAC1 activity on various concentrations of cAMP, I942 and I178 was analysed (Fig. 4c). Quasi-saturation was reached for both compounds and AC_50_ values of 40 μM, 50 μM and 200 μM were determined for cAMP, I942 and I178, respectively. The AC_50_ values are basically identical to the K_d_ under the chosen experimental conditions^29^. Thus cAMP and I942 bind EPAC1 with approximately the same affinity, which is greater than that of I178. However, the maximal activity induced by I942 is around 10 fold lower than that of cAMP (Fig. 4c). In agreement with this, I942 and I178 inhibits cAMP-induced activation of EPAC1 in a concentration dependent manner (Fig. 4c; right panel). In the presence of 500 μM cAMP a half maximal inhibition is observed at about 300 μM I942, which is roughly at equimolar concentrations. This data strongly suggest that I942 and cAMP compete for binding to the CNB domain with similar affinities.

**Figure 5:**
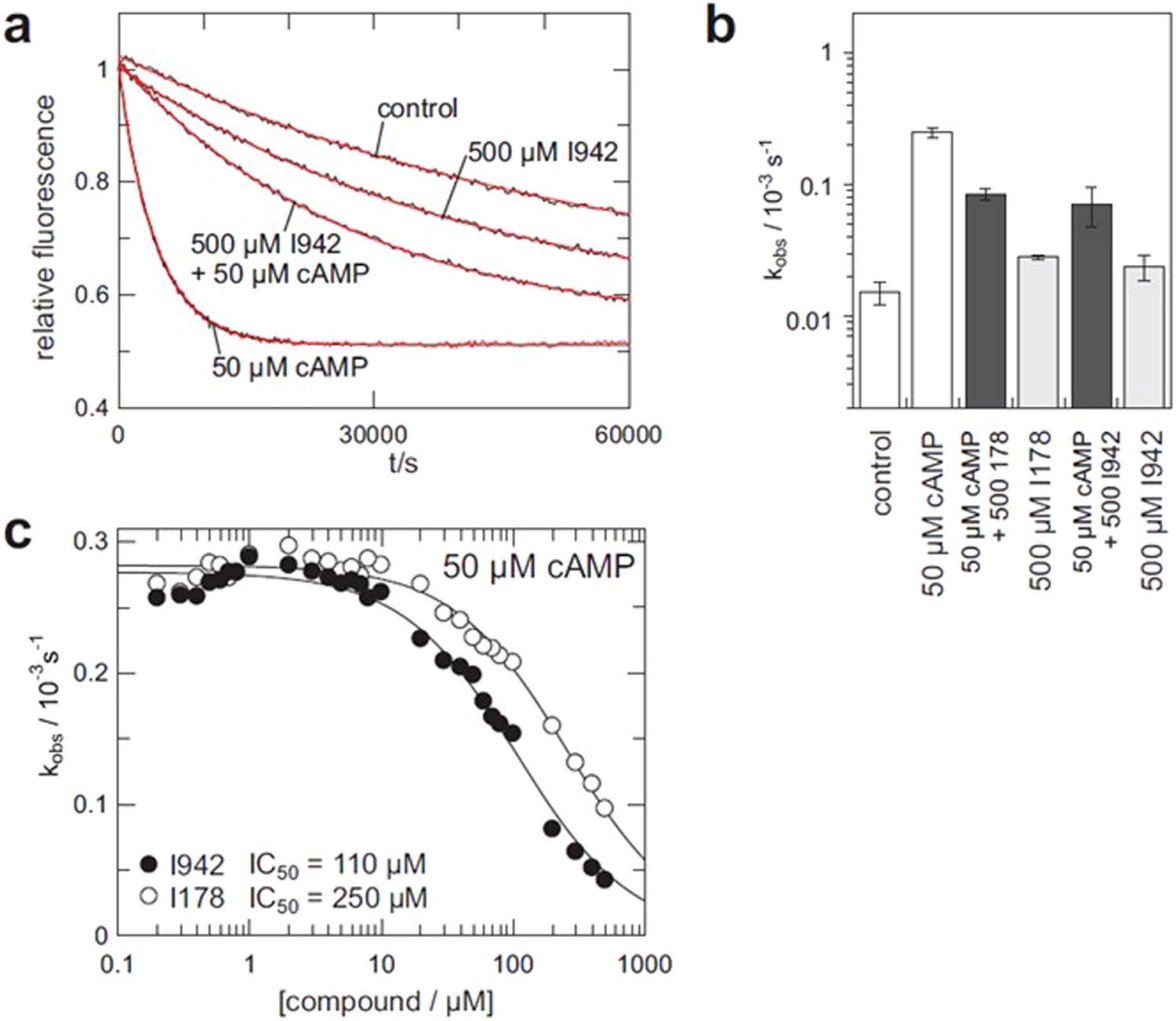
I942 and I178 have reduced Agonist Potential against EPAC2 Compared with EPAC1. (**a**) Time trace of the exchange reaction with recombinant EPAC2^280–993^ with cAMP (50 μM), I942 (500 μM) or a combination of cAMP (50 μM) and I942 (500 μM). (**b**) Rate constants from exchange reactions (n = 4)in the presence of I942 (500 μM), I178 (500 μM) and/or cAMP (50 μM). (**c**) Inhibition of cAMP induces exchange activity of Epac2^280–993^. k_obs_ were determined in the presence of 50 μM cAMP and various concentrations of I942 and I178.

We next sought to assess whether I942 and I178 also regulate EPAC2 activity and found that I942 and I178 inhibited cAMP induced activation of EPAC2^280–993^, with I942 being the most potent inhibitor (Fig. 5). The affinity of Epac2^280–993^ for cAMP is 2 μM^26, 30^, roughly 10 times higher than that of EPAC1. As such experiments were performed at 50 μM cAMP instead of 500 μM cAMP as was used for EPAC1 (Fig. 4). In this case, I942 and I178 induced EPAC2 activity is very weak (Fig. 5b and c). This rather small activity does not allow the determination of the affinity of I942 and I178 for EPAC2 from a titration monitoring the activation of EPAC2. However, in the presence of 50 μM cAMP, I942 inhibits Epac2^280–993^ with an IC_50_ of about 100 μM, compared to 250 μM for I178 (Fig. 5d). This is at a concentration slightly higher than equi-molarity. Thus, the affinity of I942 for EPAC2^280–993^ might be approximated to be 5 μM. If the affinities of I942 for EPAC1 and EPAC2 are put in relation to the affinities of cAMP, one can conclude that I942 inhibits both EPAC proteins with similar efficiency. The agonist action of I942 on EPAC2 is, however, smaller than that towards EPAC1, suggesting isoform selective partial-agonism. Such differences between EPAC1 and EPAC2 are also observed with cAMP analogues^26^. The partial agonism towards EPAC1 does not appear to also extend to PKA. We measured the effects of a range of concentrations of I942 on PKA activity induced by 1 mM cAMP, in cell extracts. This was done in comparison with the general PKA-inhibitor, H-89. We found that while H-89 treatment exerted a dose-dependent inhibition of PKA in cell extracts, whereas I942 did not significantly affect PKA activity, even at a test concentration of 100 μM (Supplementary Figure 7). It appears that the actions of I942 are selective towards the CNBs of EPAC proteins.

### Verification of interaction of compounds I942 and I178 with EPAC-CNBs by Ligand Observed Nuclear Magnetic Resonance (NMR)

**Figure 6:**
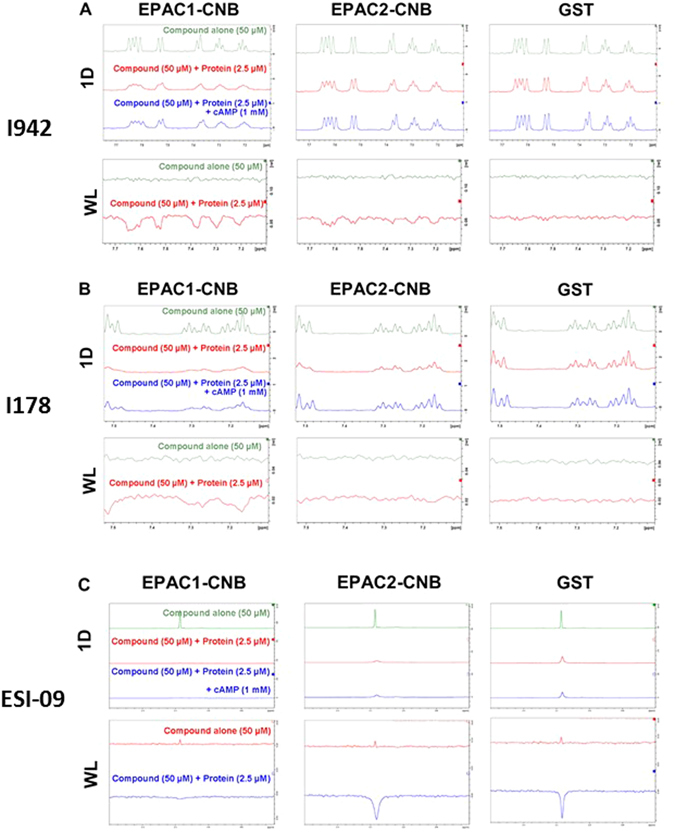
Ligand Observed NMR reveals Hit compounds Interact Directly with EPAC1-CNB and EPAC2-CNB, but not GST alone. (**A**) One dimensional proton (1D) and waterLOGSY (WL) spectra were performed on samples containing hit compound I942 (50 µM) in the absence and presence of EPAC1-CNB, EPAC2-CNB or GST alone, as indicated (2.5 µM). A representative region of the I942 spectrum is shown (7.1–7.75 ppm). Interaction between compound and protein was assessed through comparison of the compound alone and protein-compound sample spectra. The NMR data are representative of data collected at several protein and ligand concentrations. (**B**) 1D and WL spectra were performed on samples containing I178 (50 µM) in the absence and presence of EPAC1-CNB, EPAC2-CNB or GST alone, as indicated (2.5 µM). A representative region of the I178 spectrum is shown (7.1–7.51 ppm). (**C**) 1D and WL spectra were performed on samples containing the commercially available EPAC selective inhibitor ESI-09 (50 µM) in the absence and presence of EPAC1-CNB, EPAC2-CNB or GST alone, as indicated (2.5 µM). A representative region of the ESI-09 spectrum is shown (0.6–1.2 ppm).

Given that I942 seems to exert selective agonist properties against EPAC1, but not EPAC2, we next sought to validate whether our isolated ligands can interact with EPAC1 and EPAC2. Ligand observed nuclear magnetic resonance (NMR) is a valuable tool for the identification and validation of small molecule ligands^31^. We therefore used one dimensional proton (1D) and water-ligand observed by gradient spectroscopy (waterLOGSY) NMR to confirm the ability of I942 and I178 to bind to the purified CNBs of EPAC1 and EPAC2, in comparison with the commercially available EPAC1 antagonist, ESI-09^32^. In order to assess ligand/CNB interaction, 1D and waterLOGSY experiments were performed on each compound (50 µM) in the presence or absence of 2.5 µM EPAC-CNB. In the absence of protein, each test compound produced a well-resolved 1D spectrum and a region of each spectrum was chosen to display distinctive spectral peaks originating from the ligand (Fig. 6). In the absence of protein, the corresponding waterLOGSY peaks were small, or absent, due to the negligible signal produced by saturation transfer from water to the freely tumbling small molecule. In the presence of EPAC1-CNB and EPAC2-CNB the peaks originating from I942 (Fig. 6A) and I178 (Fig. 6B) became broadened, indicating that the compounds interact with the added CNBs. No peak broadening was observed for either compound in the presence of GST protein alone, indication that ligand interaction with the EPAC-CNBs was specific. In waterLOGSY spectra, protein-mediated saturation transfer can only occur if the protein-ligand complex has a substantial lifetime. Moreover, saturation transfer from ^1^H_2_O to the ligand that is mediated by the protein is of an opposing sign compared to direct transfer from water. As a result, the waterLOGSY signal obtained from a compound in the presence of a protein with which it interacts displays characteristic inverted peaks. We found this to be the case for I942 and I178 in the presence of the EPAC-CNBs, but not in the presence of GST alone (Fig. 6A and B). Interestingly, I942’s signals were broadened (1D) and inverted (waterLOGSY) in the presence of both EPAC1-CNB and EPAC2-CNB (Fig. 6A). In contrast, I178 appeared to bind selectively to EPAC1-CNB (Fig. 6B), since its 1D NMR signals were heavily broadened in the presence of EPAC1-CNB, but only slightly attenuated in the presence of EPAC2-CNBD, and it gave rise to waterLOGSY peaks with EPAC1-CNB, but not with EPAC2-CNB.

In order to test the manner in which each compound interacts with the EPAC-CNBs, 1D and waterLOGSY experiments were performed on ligand (50 µM)/protein (2.5 µM) complexes in the presence of saturating levels of cAMP (1 mM). Consistent with competition observed in the presence of cAMP during HTS, the 1D peak broadening associated with compound-protein interaction was partially reversed in the presence of saturating concentrations of cAMP (Fig. 6). This reversibility indicates that cAMP is able to displace I178 or I942 from interacting with the CNB and that the displacement of 8-NBD-cAMP by I178 and I942 seen in HTS (Fig. 3) is unlikely to occur as a result of protein denaturation or other, non-specific effects. No reversal of the waterLOGSY peaks was observed in the presence of saturating levels of cAMP, likely due to the long mixing times employed by this technique and fast kinetics of binding (data not shown). In addition, interaction between the commercially available EPAC inhibitor, ESI-09, and EPAC1-CNB, EPAC2-CNB or GST alone (Fig. 6C) was tested. We found that 1D ESI-09 peak intensity was attenuated in the presence of EPAC1-CNB, EPAC2-CNB and GST alone, indicating that ESI-09 may interact non-specifically with GST. The absence of an inverted ESI-09 peak in the presence of EPAC1-CNB can be attributed to compound binding with affinities beyond the threshold observable by waterLOGSY (sub-μM). Therefore, we conclude that both I178 and I942 interact with the EPAC1 CNB, with I178 displaying greater selectivity for EPAC1 over EPAC2. Moreover, the commercially available compound, ESI-09, induces effects at lower concentrations than I178 or I942, but does appear to make non-specific protein interactions. This is in agreement with the reported therapeutic window of ESI-09, where non-specific effects were observed at high concentrations^33^.

## Discussion

We have demonstrated that an 8-NBD-cAMP HTS competition assay can be performed using the isolated CNB of EPAC1. By optimising buffer conditions, incubation times and the concentrations of EPAC1-CNB and 8-NBD-cAMP in the assay, we were able to improve the sensitivity and power of the EPAC1-CNB assay for HTS. Subsequently, HTS of compound libraries identified two partial agonists towards EPAC1, I942 and I178. Such activation behaviour is also observed with several cAMP-analogues and is related to the activation mechanisms of EPAC^26, 34^. The core of the CNB domain blocks the access of Rap GTPases to the catalytic site in the auto-inhibited state of EPAC, which is referred to as the closed state^35^. cAMP is able to bind the CNB domain in the closed state, mainly through interactions with its phosphate sugar moiety. This initial binding causes small conformational rearrangements within the core of the CNB domain while EPAC remains in the auto-inhibited closed state. However, the core of the CNB domain is now free to tumble relative to the remaining part of the protein. This allows EPAC to adopt its open active sate. In the active state, the catalytic site is freely accessible to Rap GTPases. The CNB domain is localised at the “back” of the protein. The base of cAMP forms additional interaction with the “back” and thereby traps the core of the CNB domain at its position in the open active state^36^. Thus, in fact EPAC exists in equilibrium between inactive closed and active open conformations. This equilibrium is shifted to the active side upon binding of an agonist. The ability of the agonist to induce the conformational rearrangements in the core of the CNB domain, as a requirement for the movement of the domain, and to interact with the “back” of the protein, to stabilise the open active state, determines the extent to what the conformational equilibrium is shifted to the active side. Partial agonists are less efficient than cAMP in shifting the conformational equilibrium.

This also explains the apparent discrepancy regarding the affinity of I942 determined in the competition assay used for HTS (Fig. 3) and the activation assay (Fig. 4). The competition assay was performed with the isolated CNB domain. Binding of cAMP to the isolated CNB domain reflects only the initial binding of cAMP in the inactive closed conformation. The interactions of cAMP with the “back” are not present in the isolated CNB domain. Likewise, contributions of the re-localisation of the CNB domains to the change in the Gibbs free energy are lacking. The affinities of the isolated CNB domain and of full EPAC1 were therefore found to be 4 μM and 40 μM, respectively^30, 37^. In the competition assay the affinity of I942 was found to be about 10 fold lower than that of cAMP, and thus to be about 40 μM. In the activation assay, the affinity of I942 was determined to be 50 μM and to be roughly the same as that of cAMP. I942 therefore binds to the isolated CNB domain and to full-length EPAC1 with very similar affinities. Since I942 is a poor agonist, EPAC1 stays mainly in the closed conformation upon I942 binding and, therefore, the isolated CNB domain reflects this binding mode of I942 well. The partial agonist properties of I942 and I178 described here provide proof of concept that non-cyclic nucleotide EPAC agonists can now begin to be developed as tool molecules with the potential for future development into therapeutic agents.

## Methods

### Materials

Forskolin, rolipram, MG132 and cAMP were purchased from Merck-Millipore. Analogues of cAMP, 8-NBD-cAMP, 8-CPT-cAMP (8-CPT) and 8-pCPT-2′-O-Me-cAMP (007) were purchased from Biolog Life Sciences Institute (Bremen, Germany). The pGEX-6P-1 constructs, EPAC1-CNB and EPAC2-CNB, were prepared by Dundee Cell Products (Dundee, Scotland). BL-21 cells were purchased from New England Biolabs. The test compounds I288 (N-[(4-chlorophenyl)sulfonyl]-2-(1-naphthyloxy)acetamide) and I516 (2-[3-(acetylamino)phenoxy]-N-[(4-methylphenyl)sulfonyl]acetamide) were purchased from ChemBridge Corporation (San Diego, CA, USA), whereas I178 (N-(benzenesulfonyl)-2-(naphthalen-2-yloxy)acetamide) and I942 (N-(2,4-dimethylbenzenesulfonyl)-2-(naphthalen-2-yloxy)acetamide) were sourced from MolPort (Riga, Latvia).

### Recombinant Protein purification

EPAC1-CNB (amino acids 169–318 of EPAC1) and EPAC2-CNB (amino acids 304–453 of EPAC2) cDNAs were sub-cloned into the multi-cloning site of the pGEX-6P-1 expression vector (Invitrogen). Recombinant GST-fusion proteins were then expressed and purified from BL-21 *Escherichia coli*, as previously described^34, 38^. The purification of recombinant EPAC1 (149–881), EPAC2 (280–993) and Rap1b (1–167) from bacteria was done as previously described^29^. Protein concentrations were determined by the bicinchoninic acid (BCA) assay or absorbance at 280 nm. All recombinant proteins were determined to be pure by SDS-PAGE analysis. Proteins were stored in aliquots at −80 °C until required.

### 8-NBD-cAMP Competition Assay

Optimisation of 8-(2-[7-nitro-4-benzofurazanyl] aminoethylthio) adenosine-3′,5′-cyclic monophosphate (8-NBD-cAMP) competition assay was performed with purified EPAC1-CNB and EPAC2-CNB GST-fusion proteins, using modified assay conditions that were previously described^21^. Optimisation of assay conditions was performed in 96 well black assay plates (Greiner). Comparison of reference molecules, dose response curves and pilot screen were done in black, low volume 384 assay plates (Greiner). Liquids were dispensed using a Biomek Fx laboratory automation workstation with a 96-multichannel pipetting head (Beckman Coulter) for optimisation, whereas the Echo® Liquid Handler (Labcyte) and Wellmate automated dispenser (Thermo Matrix) were employed for pilot screens (see library screening). Plates were incubated for four hours before 8-NBD-cAMP fluorescence intensity at 480/535 nm (ex/em) was measured using the Envision multi-label plate reader (Perkin-Elmer).

### Compound Library Screening

A customised selection of structurally diverse drug-like compounds were obtained from the BioAscent Compound Cloud (www.bioascent.com/compoundcloud/, BioAscent, Biocity Scotland, Newhouse, Scotland) and were incubated with EPAC1-CNB to test for their ability to compete with 8-NBD-cAMP binding to the CNB. This was done using the optimised assay conditions developed during the study. Library compounds were transferred from pre-prepared, 1000x stock Echo® 384 LDV source plates (Labcyte) into black, low volume 384 assay plates (Greiner) at 25 nl volumes, using an Echo® Liquid Handler (Labcyte), maintaining 0.1–1% DMSO throughout. EPAC1-CNB (0.8 µM) was immediately added using a Wellmate automated dispenser (Thermo Matrix). Plates were centrifuged at 800 rpm, 10 s, and then incubated for 30 minutes, RT, to promote compound-protein complex formation. 8-NBD-cAMP was then added (62.5 nM) and the plates were then briefly centrifuged (800 × g for 10 seconds) to mix reagents. The assay was incubated at room temperature and the fluorescence intensity at 480/535 nm (ex/em) was measured after 4–15 hours using the Envision plate reader.

### Nuclear Magnetic Resonance (NMR)

All NMR spectra were recorded on a Bruker AVANCE IIIHD 600 spectrometer equipped with a TCI cryoprobe at 285 K. Test compounds were prepared in deuterated DMSO and diluted to given experimental concentrations in NMR buffer (20 mM Na_2_PO_4_, pH 7.0, 50 mM NaCl, 0.1% (w/v) NaN_3_, 5% (v/v) D_2_O) with a final DMSO concentration below 0.1%. Stocks of cAMP and recombinant proteins were prepared in NMR buffer. One dimensional proton (1D) and waterLOGSY spectra were recorded for all compounds tested, using pulse sequences as previously described^39^. Data was processed using Bruker Topspin software.

### *In Vitro* Guanine Nucleotide Exchange Factor (GEF) Activity Assay

EPAC1 and EPAC2 *in vitro* GEF activities were calculated as previously described^29^. Briefly, recombinant EPAC1 (amino acids 149–881) or EPAC2 (amino acids 280–993) were incubated at 100 nM with recombinant Rap1 (200 nM) preloaded with the fluorescent GDP analogue 2′/3′-O-(N-Methylanthraniloyl)guanosine 5-diphosphate (MANT-GDP) in the presence of 20 μM GDP and then the fluorescence intensity was measured at 360/450 nm (ex/em) over time. Multiple reactions were performed at cAMP/compound concentration varying between 0.1 and 10000 μM and curves were fit as single exponential decay to obtain k_obs_. AC_50_ or IC_50_ values were determined from the plots of k_obs_ against the concentration of the agonist and antagonist, respectively Table 1.

**Table 1:** Summary of I942 and I178 activities *in vitro.*

Experimental Techniques	EPAC1		EPAC2	
	I942	I178	I942	I178
8NBD-cAMP Competition Assays	IC_50_ ≈ 35 μM	IC_50_ ≈ 40 μM	IC_50_ ≈ 35 μM	IC_50_ ≈ 40 μM
GEF Activation (no cAMP)	AC_50_ ≈ 50 μM	AC_50_ ≈ 200 μM	N/A	N/A
GEF Inhibition (with cAMP)	IC_50_ ≈ 300 μM	IC_50_ ≈ 500 μM	IC_50_ ≈ 110 μM	IC_50_ ≈ 250 μM
NMR	Strong, Specific Interaction	Strong, Specific Interaction	Strong, Specific Interaction	Weak, specific interaction

The table shows the relative activities of I942 and I178 against EPAC1 and EPAC2 in NMR, CNBD binding assays (8NBD-cAMP Competition Assays) and *in vitro* GEF activity assays, in the presence or absence of cAMP. IC_50_ refers to the ability of I942 and I178 to compete with 8NBD-cAMP binding to isolated CNBs or inhibit EPAC GEF activity in the presence of cAMP. AC_50_, refers to the ability of I942 and I178 to promote EPAC GEF activity in the absence of cAMP. N/A refers to the inability of I942 and I178 to promote GEF activity of EPAC2.

### Protein Kinase A (PKA) Assay

PKA activity was measured directly in cell extracts using the PKA Kinase Activity Assay Kit (Abcam) following the manufacturer’s instructions. Whole cell extracts were prepared from HEK293T cells stably expressing full-length human EPAC1^40^ using Nuclear Extract Kit from Active Motif. Cells were washed in ice-cold PBS containing phosphatase inhibitors, scrapped into Eppendorf tubes and centrifuged at 200 × g at 4 °C for 5 minutes. Supernatants were discarded and the cell pellet was re-suspended in Complete Lysis Buffer containing 1 mM DTT, 1 mM PMSF and protease inhibitor cocktail (Roche) and then incubated for 10 minutes on ice (on the rocking platform) followed by vortex and centrifugation at 13,00 × g at 4 °C for 20 minutes. Cell extracts were then transferred to fresh Eppendorf tubes, assayed for protein content and then stored at −80 °C. For PKA activity measurement, ≈2 μg of whole cell extract diluted in kinase buffer were treated with 1 µM cAMP and various concentrations (0.1 to 100 µM) of I942 or the PKA inhibitor, H-89, in an ELISA plate coated with PKA substrate peptide. Next, 10 μl of ATP was added to each well and incubated at 30 °C for 90 min, with gentle shaking. The reaction was stopped by aspirating the contents of each well and then 40 µl of PKA phosphospecific substrate antibody was added to each well and then the plate was covered with plastic wrap and incubated at room temperature for 60 min (with gentle shaking every 20 min). Wells were rinsed 4 times with 1 x wash buffer (100 µl) and, after the final wash, the plate was inverted and dried on a clean paper towel to remove any remaining wash buffer. Anti-Rabbit IgG:HRP Conjugate (1:1000) was then added to each well and incubated at room temperature for 30 minutes with gentle shaking every 10 min. The wells were aspirated again and washed as described above. Next, 60 μl of the HRP substrate was added to each well and incubated for 30 minutes at room temperature. Stop solution was then added and the absorbance was measured in a plate reader at 450 nm.

### Statistical Analyses

Statistical significance was determined using one-way analysis of variance (ANOVA) with Tukey post-tests. The coefficient of variance (%CV) for assays was calculated by dividing the standard deviation of a given response by the mean signal intensity and then multiplying by 100. The signal to background ratio (S/B) was determined by dividing the mean maximum fluorescent signal (DMSO diluent control) by the mean minimum signal (saturating cAMP concentrations) to give the magnitude of the fluorescence change in the assay. The Z’ factor was also calculated from the minimum (***n***, cAMP) and maximum (***p***, DMSO) control values from the assay, together with the mean fluorescent values (***μ*** **)** and their standard deviation (***σ***), using the following equation:

$${\boldsymbol{Z}}^{\prime} {\boldsymbol{=}}1{\boldsymbol{-}}\frac{{\boldsymbol{(}}3{\boldsymbol{\sigma }}p{\boldsymbol{+}}3{\boldsymbol{\sigma }}n)}{{\boldsymbol{(}}{\boldsymbol{\mu }}p{\boldsymbol{-}}{\boldsymbol{\mu }}n{\boldsymbol{)}}}$$

## Electronic supplementary material

Supplementary Figures
