# Supplementary material for: Identification of a Novel, Small Molecule Partial Agonist for the Cyclic AMP Sensor, EPAC1
Source: Sci Rep. 2017 Mar 22;7:294. doi: 10.1038/s41598-017-00455-7 (PMC5428521; doi:10.1038/s41598-017-00455-7)

Supplementary Materials

Identification of a Novel, Small Molecule Partial Agonist for the Cyclic AMP Sensor, EPAC1 - Euan Parnell, Stuart P. McElroy, Jolanta Wiejak, Gemma L. Baillie, Alison Porter, David R. Adams, Holger Rehmann, Brian O. Smith, Stephen J. Yarwood

Supplementary Figure 1 Purification of EPAC1 and EPAC2-CNBs

Analytical gels of samples taken during the purification of EPAC1-CNB (*left*) and EPAC2-CNB (*right*) show the successful induction (with 1 mM IPTG) and purification of a ~50 kDa band (arrow) consistent with purified EPAC-CNBs.

Supplemental Figure 2 Effects of Varying Assay Parameters on the Fluorescence of 8-NBD-cAMP/EPAC1-CNB Binding.

a) EPAC1-CNB (0.8 µM) was incubated in the presence of 65.5 nM 8-NBD-cAMP, as described in materials and methods, in assay buffer with varying pH and salt (NaCl) concentrations, as indicated. The fluorescence intensity is expressed relative to the fluorescence of 8-NBD-cAMP, in the absence of protein and is expressed as a percentage increase compared to the fluorescence intensity obtained under initial buffer conditions (150 mM salt, pH 7.5). The shading scale proceeds from light to dark as fluorescence intensity increases.

b) Varying concentrations of EPAC1-CNB (protein) were incubated with a range of 8-NBD-cAMP (probe) concentrations, as indicated, using the optimised buffer conditions determined in Supplemental Figure 1a. The signal to background ratio (S/B) for the assay was then calculated. The dotted line indicates a minimal three fold S/B threshold desired for HTS.

Supplementary Figure 3 Effects of Varying Time and DMSO Concentration on the Fluorescence of EPAC1-CNB/8-NBD-cAMP Binding

a) EPAC1-CNB was incubated for the indicated times in the presence of 8-NBD-cAMP under the optimised assay conditions described in Supplementary Figure 1. Fluorescence intensity was then measured as described in materials and methods and presented as mean ± interquartile range (n=4).
b) EPAC1-CNB was incubated with 8-NBD-cAMP for four hours in the presence of increasing concentrations of DMSO, as indicated, after which the fluorescence intensity was measured as described in materials and methods.

Supplementary Figure 4 Intraplate Variability of the Optimised 8-NBD-cAMP Assay

EPAC1-CNB was incubated for four hours in black 384 well microtiter plates, using the optimised assay conditions described in Supplementary Figure 1. Increasing concentrations of cAMP in quadruplicate were seeded into wells in two different quadrants on the plate (Quadrant 1 = A4/D4-A14/D14 and quadrant 4 = M14/P14-M24/P24). The fluorescence intensity from the two quadrants were then measured and plotted against cAMP concentration. Data is presented as mean +/- standard deviation and %CV in the table.

Supplementary Figure 5 Intraplate Variability of the Optimised 8-NBD-cAMP Assay

EPAC1-CNB was incubated for four hours in black 384 well microtiter plates, using the optimised assay conditions described in Supplementary Figure 1. Increasing concentrations of cAMP were seeded into quadruplicate wells in the same quadrant on two different plates. The fluorescence intensity from the two quadrants was then measured and plotted against cAMP concentration. Data is presented as mean +/- standard deviation and %CV in the table.

Supplementary Figure 6 I942 and I178 do not Exhibit Auto-fluorescence in the Range of Potencies used for 8-NBD-cAMP Binding Assays

Increasing concentration of I942 or I178 were mixed in a cuvette with increasing concentrations of 8-NBD-cAMP. Fluorescence was then measured at 480/530 nm.

Supplementary Figure 7 I942 does not affect PKA activity

Extracts were prepared from HEK293 cells and then incubated with incubated with 1mM cAMP in the presence or absence of the indicated concentrations of I942 or the PKA inhibitor, H-89, as described in Materials and Methods. PKA activity was then measured in the cell extracts using an ELISA assay according to the manufactures instructions. The graph is plotted as means±SD for three observations.

Supplementary Figure 1

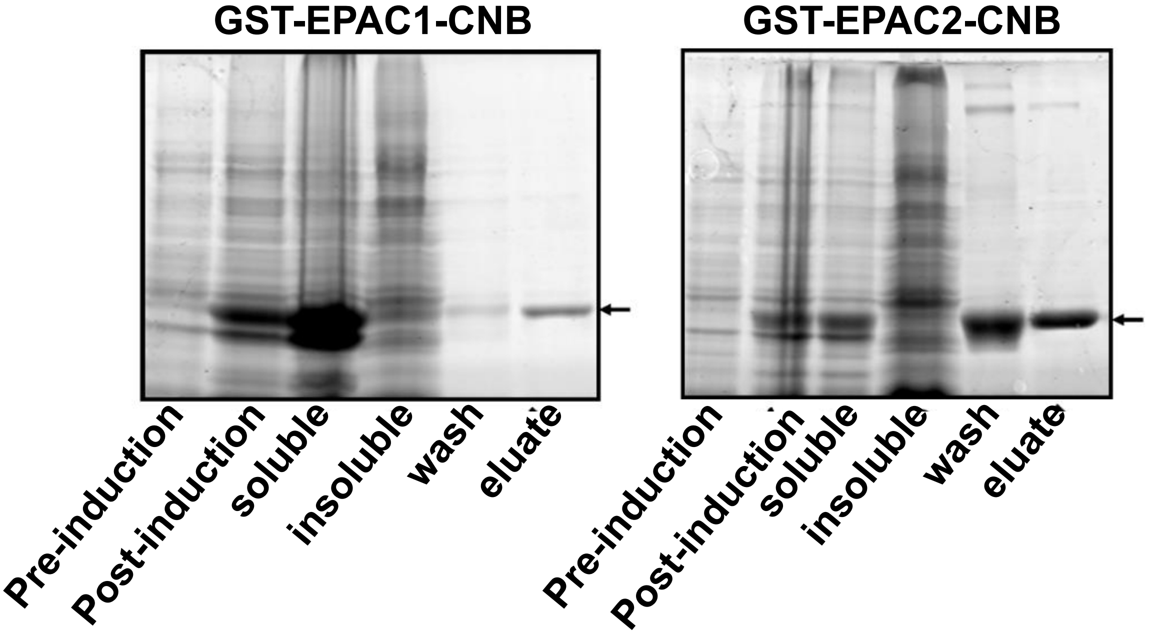

Supplementary Figure 2

A)

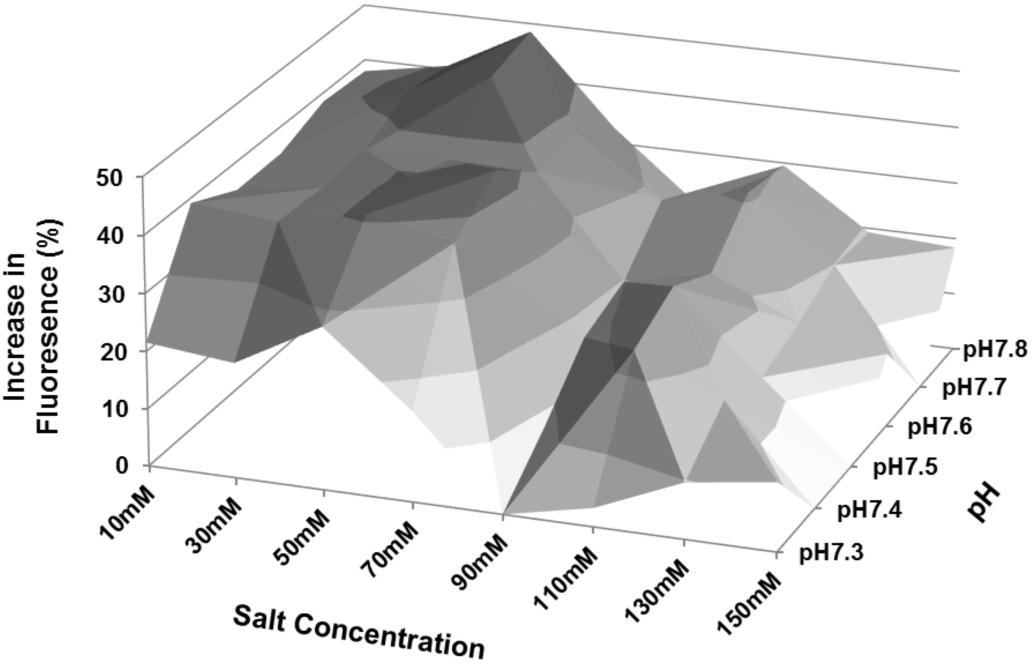

B)

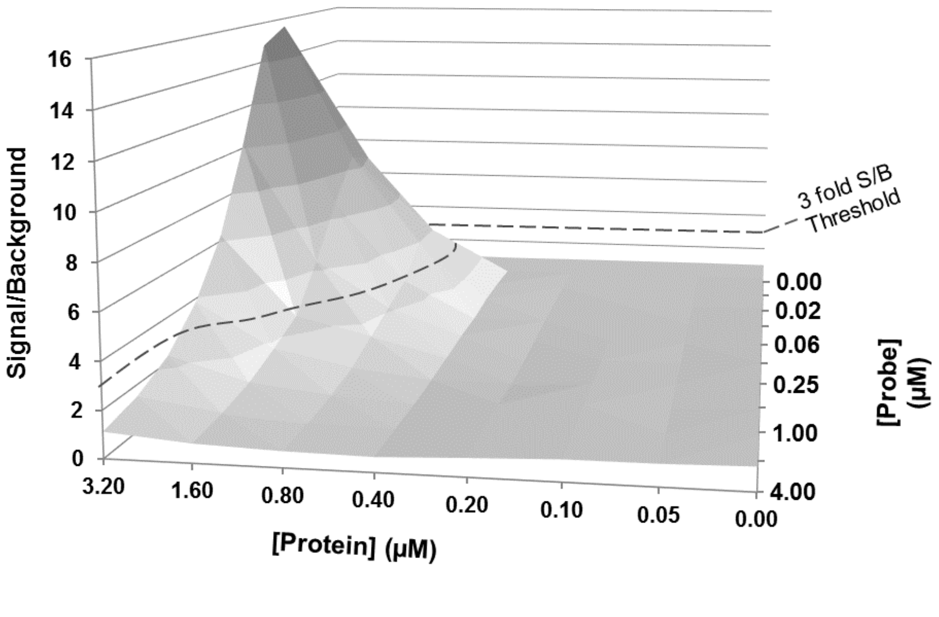

Supplementary Figure 3

A)

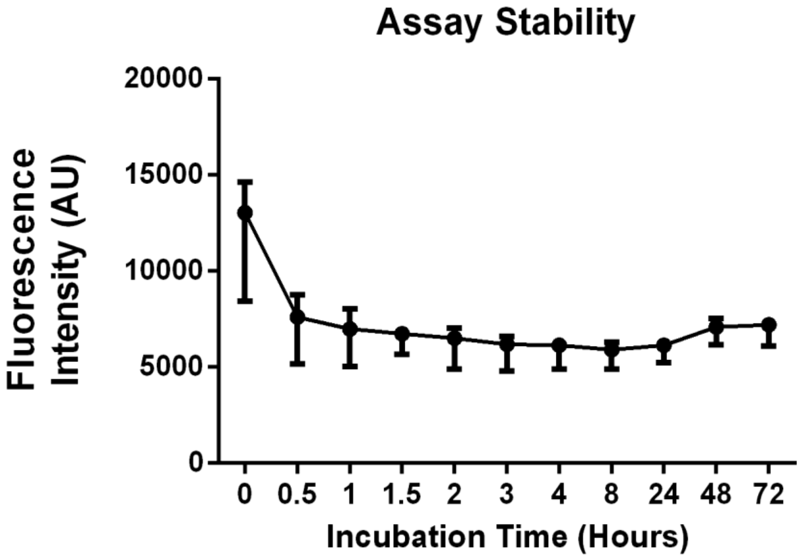

B)

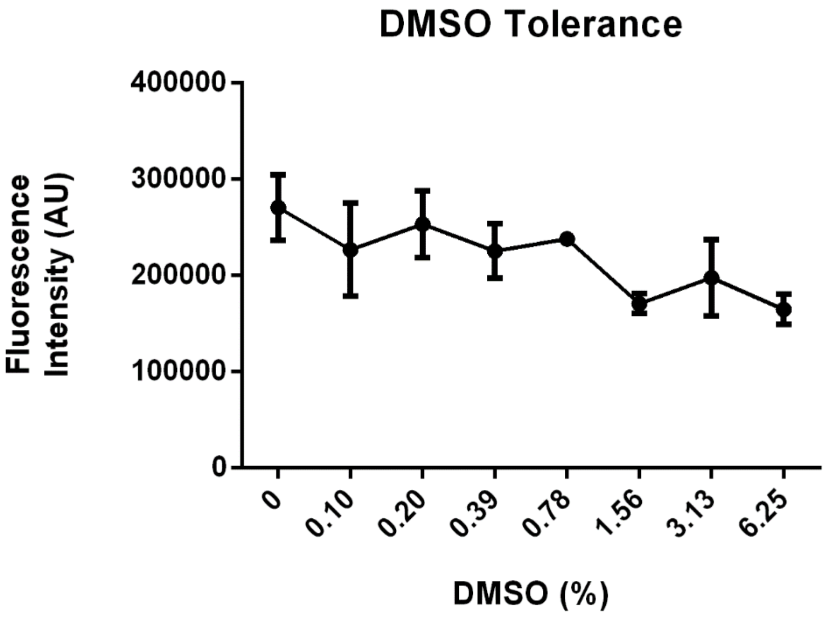

Supplementary Figure 4

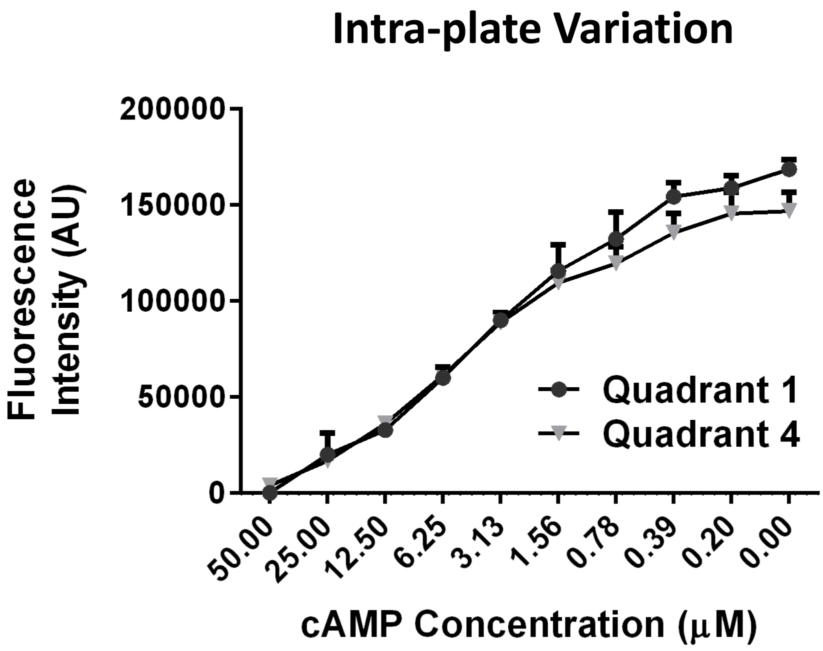

Intra-plate	Mean ± SD	%CV	Intra-plate	Mean ± SD	%CV
**Max 1**	203940 ± 12608	6.2	**Max 1 vs Max 2**	206804 ± 4051	1.9
**Max 2**	209669 ± 16884	8.1			
**Min 1**	43206 ± 4345	10.1	**Max 1 vs Max 2**	42949 ± 363	0.9
**Min 2**	42693 ± 2918	6.8			

Supplementary Figure 5

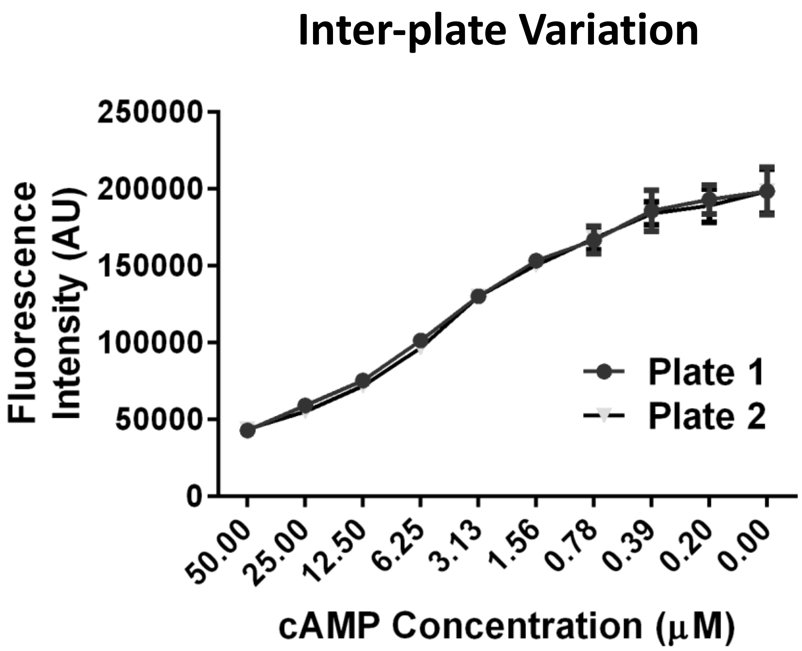

Inter-plate	Mean ± SD	%CV	Inter-plate	Mean ± SD	%CV
**Max (plate 1)**	199791 ± 5866	2.9	**Plate 1 vs Plate 2**	199492 ± 424	0.2
**Max (plate 2)**	199192 ± 14817	7.4			
**Min (plate 1)**	43704 ± 703	1.6	**Plate 1 vs Plate 2**	43200 ±713	1.7
**Min (plate 2)**	42696 ± 4	0.1			

Supplementary Figure 6

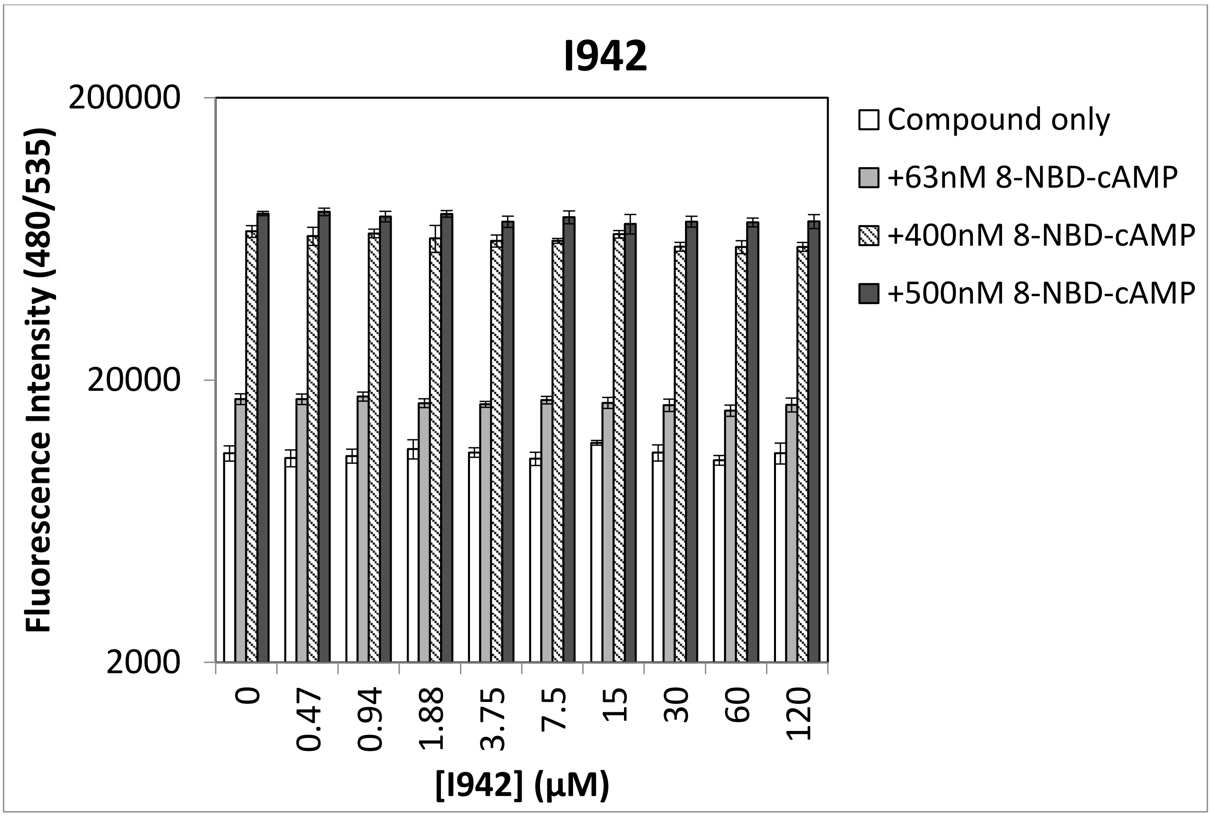

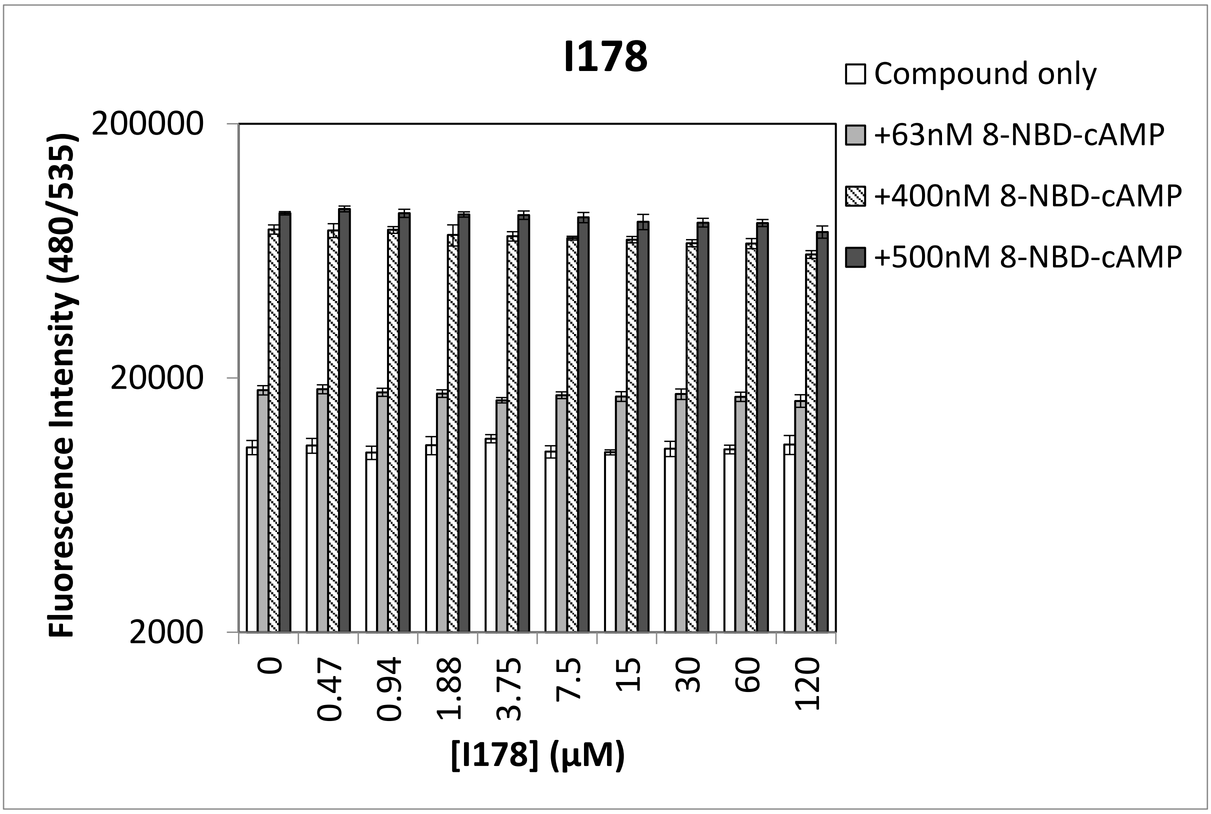

Supplementary Figure 7

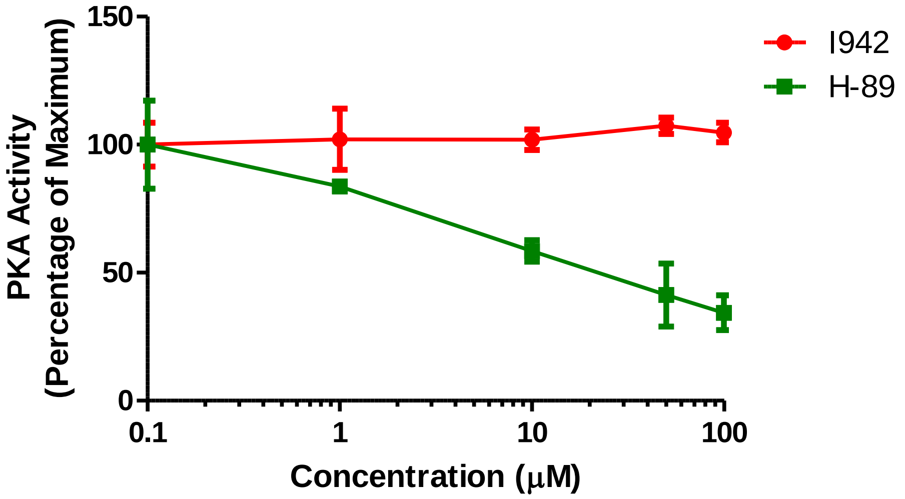

Supplement: Supplementary file 1 — Supplementary Figures [file 41598_2017_455_MOESM1_ESM.pdf]
